# Premorbid functional status as an outcome predictor in intensive care patients aged over 85 years

**DOI:** 10.1186/s12877-021-02746-1

**Published:** 2022-01-10

**Authors:** Laura Pietiläinen, Minna Bäcklund, Johanna Hästbacka, Matti Reinikainen

**Affiliations:** 1 University of Eastern Finland and Department of Anaesthesiology and Intensive Care, Kuopio University Hospital, Kuopio, Finland; 2 Division of Intensive Care Medicine, Department of Anaesthesiology, Intensive Care and Pain Medicine, University of Helsinki and Helsinki University Hospital, Helsinki, Finland

**Keywords:** Aged over 85, Intensive care, ICU, Outcome, Functional status, Frailty

## Abstract

**Background:**

Poor premorbid functional status (PFS) is associated with mortality after intensive care unit (ICU) admission in patients aged 80 years or older. In the subgroup of very old ICU patients, the ability to recover from critical illness varies irrespective of age. To assess the predictive ability of PFS also among the patients aged 85 or older we set out the current study.

**Methods:**

In this nationwide observational registry study based on the Finnish Intensive Care Consortium database, we analysed data of patients aged 85 years or over treated in ICUs between May 2012 and December 2015. We defined PFS as good for patients who had been independent in activities of daily living (ADL) and able to climb stairs and as poor for those who were dependent on help or unable to climb stairs.

To assess patients’ functional outcome one year after ICU admission, we created a functional status score (FSS) based on how many out of five physical activities (getting out of bed, moving indoors, dressing, climbing stairs, and walking 400 m) the patient could manage. We also assessed the patients’ ability to return to their previous type of accommodation.

**Results:**

Overall, 2037 (3.3% of all adult ICU patients) patients were 85 years old or older. The average age of the study population was 87 years. Data on PFS were available for 1446 (71.0%) patients (good for 48.8% and poor for 51.2%). The one-year mortalities of patients with good and those with poor PFS were 29.2% and 50.1%, respectively, *p* < 0.001. Poor PFS increased the probability of death within 12 months, adjusted odds ratio (OR), 2.15; 95% confidence interval (CI) 1.68–2.76, *p* < 0.001. For 69.5% of survivors, the FSS one year after ICU admission was unchanged or higher than their premorbid FSS and 84.2% of patients living at home before ICU admission still lived at home.

**Conclusions:**

Poor PFS doubled the odds of death within one year. For most survivors, functional status was comparable to the premorbid status.

**Supplementary Information:**

The online version contains supplementary material available at 10.1186/s12877-021-02746-1.

## Background

The need to make decisions about admitting very old (aged 80 years or older) patients to the intensive care unit (ICU) increases as the general life expectancy increases. In the last decade, there has been an increasing interest in the ICU outcomes of the oldest old people, namely, patients aged 85 years or older and even patients aged 90 years or older [1, 2].

These patients may have an impaired capacity to recover from a critical illness [3–6], reflected in the ICU prognostic scoring systems, where all patients aged 80 years or older equally receive a maximal number of points from age [7, 8]. However, in the subgroup of very old ICU patients, the ability to recover from critical illness varies irrespective of age [9–11]. Therefore, focusing on factors reflecting the physiological potential for recovery could be helpful in decision-making. Examples of such factors are functional capacity and frailty, which have been documented in several ICU studies and found to be useful in predicting outcomes in very old intensive care patients [12–16].

In our previous study, we found that a poor premorbid functional status (PFS) [needing assistance for activities of daily living (ADL) or being unable to climb stairs] is associated with a twofold increase in the odds of death within 12 months after ICU admission in patients aged 80 years or older [17]. We also found that 78% of survivors recovered to a functional status comparable to their premorbid situation. In that study, the majority of the study population was 80 to 85 years old.

We designed the current study to assess the predictive ability of PFS among the oldest old. We aimed to assess 1) PFS and its association with one-year outcome of ICU patients aged 85 years or older and 2) to study their functional recovery by using a functional severity score and ability to return to live at home. We hypothesized that poor PFS would be associated with poor 1-year outcome also in this oldest old ICU population.

## Methods

This is an observational registry study based on the nationwide Finnish Intensive Care Consortium’s (FICC) database. We included data on admissions of the oldest old patients (those aged 85 or older) from May 2012 to December 2015. We excluded readmissions but had no other exclusion criteria. A part of the current study population (patients admitted between May 2012 and April 2013) made up the study population in our previous study [17].

The FICC’s database includes detailed information about patient characteristics, physiological variables, premorbid functional status and severity of illness of patients admitted to general adult ICUs in Finland [18]. We gathered data on the baseline characteristics: age, sex, type of ICU admission (scheduled surgical, emergency surgical or medical), diagnostic category (cardiac or vascular surgery, gastrointestinal surgery, neurological or neurosurgical diseases, trauma, other surgery, cardiovascular diseases, respiratory diseases, metabolic disturbances, intoxication and miscellaneous), length of stay (LOS) and treatment restrictions applied in the ICU.

In the FICC database, severity of illness is measured using the Simplified Acute Physiology Score II (SAPS II) [8], occurrence and severity of organ dysfunctions during the first 24 h are measured using the Sequential Organ Failure Assessment (SOFA) [19, 20]. Treatment intensity is measured using the Therapeutic Intervention Scoring System 76 (TISS-76) [21], that represents the intensity of care based on the work load and therapeutic interventions used (for example mechanical ventilation, vasoactive treatment, invasive monitoring and haemodialysis).

The premorbid status data of the ICU patients routinely recorded include the physical performance according to the WHO/ECOG performance status classification [22], patient´s ability to manage five physical activities (getting out of bed, moving indoors, dressing, climbing stairs, and walking 400 m) [23, 24] and current accommodation type (living at home or in institutional care). We utilized WHO/ECOG data to assess independence in ADL and data on the five functional status variables regarding the patients’ ability to manage without assistance. Based on findings of our previous study [17], we defined the premorbid functional status as good for those able to climb stairs without assistance and independent in ADL Conversely, we defined PFS as poor for those who were unable to climb stairs without assistance or were dependent on assistance in ADL. [17] We then compared mortality outcomes between patients with good and poor PFS.

In addition to mortality, we investigated functional recovery among survivors by comparing premorbid status with the functional status one year after admission. To allow comparison, we created a functional status score (FSS), based on the number of manageable activities (out of the five) the patient was able to perform without assistance. We obtained the one-year functional performance data from a written questionnaire that was sent to all survivors.

Our primary endpoints were the association of PFS with one-year mortality and functional status one year after intensive care using the FSS. Returning to premorbid accommodation type, hospital mortality, intensity of treatment and decisions to restrict treatment activity were secondary endpoints.

We used SPSS version 27 (IBM Corp, Armonk, NY, USA) in our statistical analyses. We considered a p value lower than 0.05 as statistically significant. Data for continuous variables are reported as median values and interquartile ranges (IQR); data for categorical variables are presented as numbers of cases and percentages. A chi-square test was used to compare the categorical variables and the Mann–Whitney *U* test for continuous variables. We performed univariable and multivariable regression analyses to assess the association of baseline variables and poor premorbid functional status with outcome. We present all results of univariable and multivariable analyses as odds ratios (OR) with 95% confidence intervals (CI).

We calculated the predicted probabilities of one-year mortality with two models. The first model included age, sex, admission type and SAPS II score without admission type points. In the second model, we included PFS in addition to the variables in the first model. We assessed the Area Under the Receiver Operating Characteristic (AUROC) curve for each model to evaluate whether adding PFS data improves the predictive ability of the prognostic model. We tested the statistical significance of the difference between the AUROC values with R statistical software using the roc.test function in the ROC package with the paired samples option and the bootstrap method [25].

We performed another multivariable logistic regression analysis to assess the impact of age, sex, type of admission, SAPS II without admission type and premorbid functional status on orders to restrict treatment activity. An order to restrict treatments typically means a do-not-attempt-resuscitation order, but it may also mean an order to withhold some aggressive treatments (e.g. invasive mechanical ventilation or renal replacement therapy) or to withdraw ongoing treatments because of presumed futility.

## Results

During the study period from May 2012 to December 2015, there were 65,444 admissions of patients aged 18 years or older. After excluding readmissions during the same hospitalization (*n* = 3,865), there were 61,579 patients which comprised 2,037 (3.3%) patients aged 85 years old or older. The baseline characteristics and mortality outcomes of the study population are presented in Table 1. For the overall ICU patient population, hospital and one-year mortalities were 11.3% and 22.4%, respectively.

**Table 1:** Baseline characteristics and mortality outcomes of the study population

Characteristic	85 years and older
Number of admissions	2037
Age, years	87 (85–89)
Male gender, n (%)	908 (44.6)
Able to live at home, n (%)	1286 (85.0)^a^
Able to move indoors, n (%)	1397 (93.9)^b^
Able to walk 400 m, n (%)	993 (67.3)^c^
Able to climb stairs, n (%)	1001 (68.3)^d^
Able to dress themselves, n (%)	1320 (89.2)^e^
Able to get out of bed, n (%)	1386 (94.0)^f^
Independent in ADL, n (%)	1180 (59.3)^g^
Good PFS, n (%)	705 (48.8)^h^
Admission type	
Scheduled surgical	373 (18.3)
Emergency surgical	545 (26.8)
Medical admission, n (%)	1119 (54.9)
Diagnostic categories, n (%)	
Cardiac or vascular surgery	413 (20.3)
Gastrointestinal surgery	298 (14.6)
Neurological/neurosurgical diseases	159 (7.8)
Trauma	162 (8.0)
Other surgery	75 (3.7)
Cardiovascular diseases	490 (24.1)
Respiratory diseases	192 (9.4)
Metabolic disturbances	205 (10.1)
Intoxication	11 (0.5)
Miscellaneous	32 (1.6)
SAPS II	40 (32–51)
SAPS II without admission type	34 (27–45)
SOFA24	6 (4–9)
TISS	27.6 (22.5–33.6)
LOS ICU	1.22 (0.83–2.74)
Treatment restrictions, n (%)	478 (23.5)
ICU mortality, n (%)	205 (10.1)
Hospital mortality, n (%)	454 (22.3)
1-year mortality, n (%)	896 (44.0)

Data for continuous variables are presented as median values (interquartile ranges), and data for categorical variables are presented as numbers of cases (%). *SAPS II* Simplified Acute Physiology Score, *SOFA* Sequential Organ Failure Assessment score, based on the first 24 h, *TISS* mean daily Therapeutic Intervention Scoring System 76 score, *LOS ICU* length of stay (days) in intensive care unit, good *PFS* good premorbid functional status (a person who can perform ADL and climb stairs without assistance). Data available for, % ^a^74.3, ^b^73.0, ^c^72.4, ^d^72.0, ^e^72.6, ^f^72.4, ^g^97.7, ^h^71.0

PFS and mortality.

**Fig. 1:**
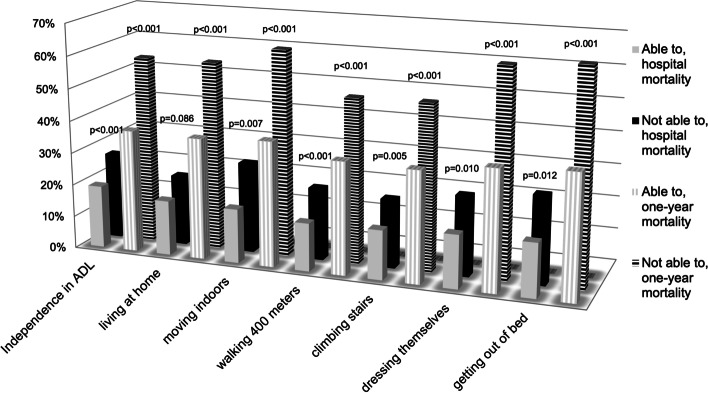
Hospital mortality and one-year mortality, according to the ability to perform physical activities. The p-values refer to comparisons between patients who could manage the activities and those who could not

Data on PFS were available for 1,446 (71.0%) patients aged 85 or older. PFS was good for 705 (48.8%) and poor for 741 (51.2%) patients. Hospital mortality was 13.0% (92/705) for patients with good PFS and 21.2% (157/741) for those with poor PFS, *p* = 0.001. One-year mortality was 29.2% (200/684) for patients with good PFS and 50.1% (355/709) for those with poor PFS, *p* < 0.001. One-year status was missing for 21 patients with good PFS and for 32 patients with poor PFS. Mortality outcomes according to each PFS component are presented in Fig. 1. The outcome in the age groups according to premorbid functional status is presented in the supplementary table 1 (Additional file 1). The baseline characteristics of patients whose PFS was not documented are presented in the supplementary table 2 (Additional file 2).

For patients aged 85 years or over, poor PFS increased the probability of death within one year, adjusted OR 2.15, 95% CI 1.68–2.76, *p* < 0.001. When PFS was added to our baseline prediction model that included age, sex, admission type and illness severity, the discriminative ability of the model improved from AUROC 0.743 [0.717–0.769] to AUROC 0.759 [0.734–0.784], *p* = 0.010. The results of the univariable and multivariable analyses of the predictors of hospital and one-year mortality are presented in Table 2.

**Table 2:** Predictors of hospital and one-year mortality in patients aged 85 years and older

**Predictors of hospital mortality**	**Univariable analysis**			**Multivariable analysis**		
	OR	95% CI	*p*- value	Adjusted OR	95% CI	*p*-value
Age^a^	1.05	1.01–1.09	0.019	1.06	1.01–1.11	0.024
Male gender^1^	1.45	1.18–1.79	< 0.001	1.25	0.97–1.61	0.079
Type of admission^2^						
Scheduled surgical	reference			reference		
Emergency surgical	7.24	4.01–13.08	< 0.001	3.88	2.09–7.19	< 0.001
Medical	11.48	6.51–20.27	< 0.001	4.73	2.61–8.57	< 0.001
SAPS II without admission type points^b^	1.09	1.08–1.10	< 0.001	1.08	1.07–1.09	< 0.001
Poor premorbid functional status^3^	1.79	1.35–2.37	< 0.001	1.57	1.13–2.19	0.007
**Predictors of one-year mortality**	**Univariable analysis**			**Multivariable analysis**		
	OR	95% CI	*p*-value	Adjusted OR	95% CI	*p*-value
Age^a^	1.10	1.06–1.14	< 0.001	1.10	1.06–1.14	< 0.001
Male gender^1^	1.23	1.03–1.47	0.022	1.12	0.91–1.37	0.275
Type of admission^2^						
Scheduled surgical	Reference			Reference		
Emergency surgical	4.66	3.33–6.51	< 0.001	2.95	2.08–4.19	< 0.001
Medical	6.96	5.10–9.50	< 0.001	4.03	2.91–5.57	< 0.001
SAPS II without admission type^b^	1.07	1.06–1.08	< 0.001	1.06	1.05–1.07	< 0.001
Poor premorbid functional status^3^	2.43	1.95–3.03	< 0.001	2.15	1.68–2.76	< 0.001

^a^For each additional year of age, ^b^for each additional point. *OR* odds ratio, *CI* confidence interval, *SAPS II* Simplified Acute Physiology Score; Poor premorbid functional status: a person dependent on assistance in activities of daily living or unable to climb stairs without assistance. The reference categories for categorical variables: ^1^female gender, ^2^scheduled surgical admission, ^3^good premorbid functional status

Functional status in one-year survivors.

**Fig. 2:**
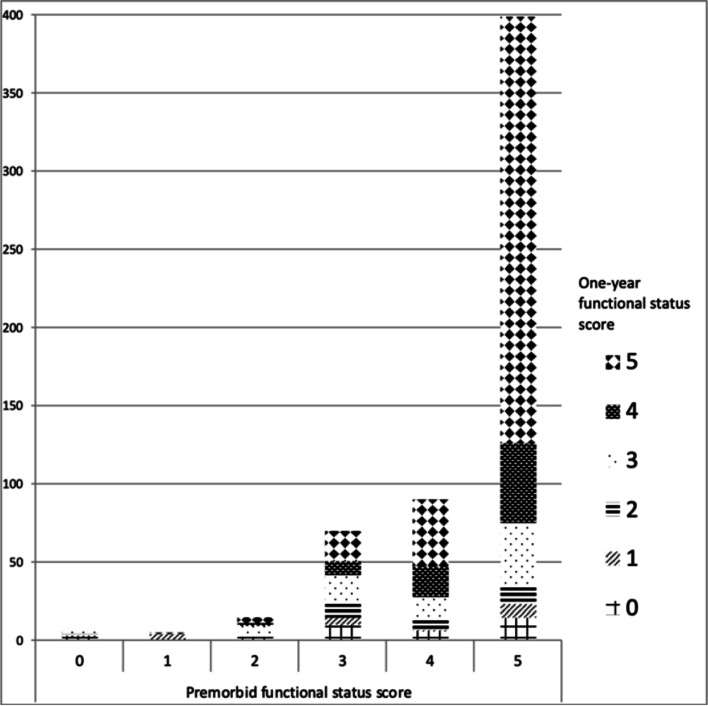
Functional status score at one year according to the premorbid functional status score. The functional status score is the number of manageable activities (getting out of bed, moving indoors, dressing, climbing stairs, and walking 400 m). The majority of patients admitted to intensive care had a premorbid functional status score between 3–5. At one year after ICU admission, most survivors had recovered to a functional status comparable to the premorbid situation. The Y axis shows the number of admissions

For one-year survivors, data on all five physical activities one year after ICU admission were available for 722/1,068 (67.6%) patients. Data on accommodation type were available for 739/1,068 (69.2%) patients. The FSS (number of manageable physical activities) was comparable to or better than that of the premorbid situation for 69.5% (408/587) of survivors one year after ICU admission (Fig. 2). Of those patients who had lived at home before the ICU admission, 84.2% (representing 78.2% of all survivors) were still living at home after one year.

Treatment restrictions and intensity.

Treatment restrictions were set for 11.6% of patients with good PFS, as compared to 28.9% of those with poor PFS (*p* < 0.001). For patients with missing PFS data, treatment restrictions were set for 30.8% of patients. In multivariable analysis [Additional file 3], emergency surgical and medical admissions, severity of illness, age and poor PFS (OR 3.09, 95% CI 2.33–4.08, *p* < 0.001) increased the probability of treatment restrictions.

Vasoactive therapy was employed for 1,442 (70.8%) patients during their ICU stay and 1,272 (62.4%) patients received mechanical ventilation. Different items of treatment intensity, based on recorded TISS items, are presented in the supplementary table 4 (Additional file 4). The intensity of treatment as measured by mean daily TISS scores was higher for patients with a good PFS than for those with a poor PFS [median 28.8 (IQR 23.2–34.5) vs. 27.3 (22.3–32.8), *p* = 0.002).

## Discussion

In this Finnish nationwide registry study involving 2,037 ICU admissions of the oldest old (those aged 85 years or older) patients, PFS was strongly associated with long-term outcome. A poor PFS doubled the odds of death within one year. Adding data on PFS to a prediction model based on age, sex, admission type and severity of illness (SAPS II score) improved one-year mortality prediction. More than half of the patients survived to one year and the functional recovery of most survivors was favourable despite advanced age.

Previously, Guidet et al. found that promoting systematic ICU admission of critically ill elderly patients, compared to usual admission practices, led to an increased ICU admission rate but did not reduce 6-month mortality. Guidet et al. concluded that the decision to admit a very old patient to the ICU should be based on the ability to identify patients who will benefit from care. [26] Our results suggest that implementing PFS in prediction models for oldest old patients could help in decision making about ICU admissions, when targeting survival and reasonable long-term recovery.

Some prediction models have been tested in previous studies to predict hospital mortality [27] and longer-term outcomes [28, 29]. The Clinical Frailty Scale (CFS) has been commonly used and for example, Flaatten et al. [12] have described CFS values of 5 or over as indicative of frailty, which is associated with worse outcomes. A CFS value less than 5 describes people who are independent and do not require help for ADL and a CFS value of 6 means having problems with stairs [30]. Our definition of poor PFS may be comparable to the definition of frailty as CFS value of 5 or higher. In recent studies, the prevalence of frailty has been found to be 43% in the very old ICU population [12] and nearly 30% in the general ICU population [14, 15]. In our study 51.2% of patients aged 85 years or older had a poor PFS.

A poor functional status is a clinical manifestation of the reduced physical and physiologic reserves including also e.g. sarcopenia which is characterised by generalized loss of skeletal muscle mass, strength and function. Recently Zhang et al. [31] have found in their systematic review and meta-analysis that the sarcopenia increases the risk of mortality in critical illness.

Many predictors were associated with both short-term outcome (hospital mortality) and longer-term outcome (one-year mortality) but the strength of association was dependent on the choice of endpoint. Severity of the acute illness and type of admission were strongly associated with hospital mortality, so that patients admitted for medical reasons or after emergency surgery had markedly higher odds of death than patients admitted after scheduled surgery. Not surprisingly, old age was a strong predictor of long-term mortality. Poor PFS was associated with increased odds of in-hospital death, but even more strongly with one-year mortality.

This study focuses on the rapidly growing oldest old (those aged over 85 years old) population. ICU admission decisions of the oldest old require careful consideration of the expected benefit to the patients. In our previous study, the majority of the elderly population was 80 to 85 years old, and to assess the predictive ability of PFS also among the oldest old (aged 85 or older) we designed the current study. These findings further confirm the importance of PFS as a determinant of long-term outcome. For most survivors, functional recovery was favourable, which is a finding of paramount importance: many patients in the group of the oldest old may benefit from intensive care, but PFS should be taken into account when making decisions about ICU admission. The meaning of these findings is that it is justifiable to include the premorbid functional status or frailty in outcome prediction models. Clinicians may benefit from such tools not only for admission decisions but also when making decisions concerning continuing or restricting ICU treatment.

This is of utmost importance, since life expectancy is increasing. Life expectancy without disabilities in ADL has also increased. Based on a Finnish population study of people aged 90 years or older, four of ten people were independent in the five activities (ability to climb stairs, to walk 400 m, to move indoors, to get in and out of bed and to dress and undress) and seven of ten people were independent in ADL and moving indoors [32]. Most probably, the usefulness of intensive care for the oldest old patients will become an increasingly common question in the future.

One year after ICU admission, 56% of our study patients aged 85 years or older were still alive, and the functional status, reflected by the number of manageable physical activities, was comparable to or better than that of the premorbid situation for seven out of 10 survivors. Eight of 10 survivors who had lived at home before the ICU admission were still living at home one year later. These results are somewhat better than those reported by Heyland et al., who found that only one quarter of ICU patients (roughly half of the survivors) aged 80 years or older had recovered to a physical function comparable to the premorbid status one year after ICU admission [33]. One plausible explanation for these differences is that we included elective surgical patients with short ICU stays in our study, whereas Heyland et al. only included patients with a LOS exceeding 24 h [33].

Treatment intensity should be based on a patient’s expected ability to benefit, and limiting aggressive treatments ideally means adjusting the intensity of care according to the patient’s capacity to recover from acute illness and benefit from ICU admission [34]. In a previous Finnish nationwide study, Reinikainen et al. found that the intensity of treatment decreased in the patients aged 80 years or older [4]. Guidet et al. [34] reported a 27.2% prevalence of treatment restrictions in their study population of very old patients. In our study, treatment was restricted in 23.5% of the patient aged 85 years old or older.

We found that poor premorbid functional status was associated with less intensive treatment and increased the probability for treatment restrictions [Additional file 3]. Likewise, Flaatten et al. [12] have found that frail patients are significantly more likely to have treatment restrictions than non-frail patients. In our study half of the patients with treatment restrictions were discharged alive from the hospital and 25% were alive one year after ICU admission, which is a proportion similar to that found in another Finnish study of treatment restrictions in the adult ICU population [35].

The major strengths of our study are the nationwide design, long-term outcome and functional recovery data covering a large number of patients. We did not exclude any admissions based on length of stay in the ICU or treatment restrictions, which increases the generalizability of our results. The data from this study include all admissions in all general adult ICUs in Finland during the study period.

Limitations of this study include its retrospective design. The ICU-treated study population is selected based on a clinical decision-making process, in which a patient’s functional capacity has most probably been considered. These practices are likely to cause selection bias towards selecting patients with better PFS and thus limits the generalizability of our results to the age groups as a whole.

Second, in our data, premorbid functional status was available only for 71.0% of patients. Collecting data on functional ability should be a routine, but often patients are not able to provide this information for example due to severity of their condition, and relatives are not always reached. Indeed, in our population severity of illness and mortality were higher for patients with missing PFS data [Additional file 2]. In addition, our data on functional status are based on self-reported information by the patient or their relatives and may thus be either underestimated or overestimated compared to objectively measured values. However, from the patients´ perspective the subjective estimation is not without importance.

## Conclusions

Good premorbid functional status, defined as independence in activities of daily living and ability to climb stairs, was associated with better odds for survival in ICU patients aged 85 years or older. For most of these oldest old ICU patients who survived one year after admission, functional status based on FSS remained comparable to the premorbid status and a majority of patients who lived at home before ICU admission still lived at home one year later. In the future, PFS may be included in outcome prediction models.

## Supplementary Information

**Additional file 1.** The outcome in the two age groups according to premorbid functional status

**Additional file 2.** Comparison of baseline characteristics and mortality outcomes of patients with documented premorbid functional status and patients with missing premorbid functional status data

**Additional file 3.** Factors predicting orders to restrict treatment activity

**Additional file 4.** Treatment intensity, based on recorded Therapeutic intervention scoring system items

## Data Availability

The data of this study were used with a personally granted license for the current study. According to the Finnish Act on the Secondary Use of Health and Social Data (552/2019), the data are not publicly available.

## Abbreviations

PFS: Premorbid functional status
ICU: Intensive care unit
ADL: Activities of daily living
FSS: Functional status score
OR: Odds ratio
CI: Confidence interval
FICC: Finnish Intensive Care Consortium
LOS: Length of stay
SAPS II: Simplified Acute Physiology Score II
SOFA: Sequential Organ Failure Assessment
TISS-76: Therapeutic Intervention Scoring System 76
IQR: Interquartile ranges
AUROC: Area Under the Receiver Operating Characteristic
CFS: Clinical Frailty Scale
