# Supplementary material for: Premorbid functional status as an outcome predictor in intensive care patients aged over 85 years
Source: BMC Geriatr. 2022 Jan 10;22:38. doi: 10.1186/s12877-021-02746-1 (PMC8751370; doi:10.1186/s12877-021-02746-1)
Supplement: Supplementary file 1 — Additional file 1. The outcome in the two age groups according to premorbid functional status [file 12877_2021_2746_MOESM1_ESM.docx]

**Supplementary table 1 The outcome according to premorbid functional status**

| **Characteristics** | **85 years and older** |  |  |
| --- | --- | --- | --- |
| **PFS data available (%)** | **1446 (71.0)** |  |  |
|  | **Good PFS** | **Poor PFS** | **p-value** |
| **Number of patients (%)** | **705 (48.8)** | **741 (51.2)** |  |
| **ICU mortality, n (%)** | **33 (4.7)** | **60 (8.1)** | **0.049** |
| **Hospital mortality, n (%)** | **92 (13.0)** | **157 (21.2)** | **<0.001** |
| **One-year mortality, n (%)** | **200 (29.2)^a^** | **355 (50.1)^b^** | **<0.001** |
| **Treatment restrictions, n (%)** | **82 (11.6)** | **214 (28.9)** | **<0.001** |
| **FSS at one year comparable or better than premorbid FSS, n (%)** | **251 (72.3)^c^** | **152 (65.2)^d^** | **0.058** |

PFS, Premorbid Functional Status; Good PFS, independence in activities of daily living (ADL) and ability to climb stairs; Poor PFS, dependency on help in ADL or unability to climb stairs; ICU intensive care unit; FSS, Functional Status Score. Data missing for ^a^21, ^b^32, ^c^358, ^d^508
