# Supplementary material for: Premorbid functional status as an outcome predictor in intensive care patients aged over 85 years
Source: BMC Geriatr. 2022 Jan 10;22:38. doi: 10.1186/s12877-021-02746-1 (PMC8751370; doi:10.1186/s12877-021-02746-1)
Supplement: Supplementary file 2 — Additional file 2. Comparison of baseline characteristics and mortality outcomes of patients with documented premorbid functional status and patients with missing premorbid functional status data [file 12877_2021_2746_MOESM2_ESM.docx]

**Supplementary table 2 Comparison of baseline characteristics and mortality outcomes of patients with documented premorbid functional status and patients with missing premorbid functional status data**

| **Characteristics** | **Premorbid functional status documented** | **Premorbid functional status missing** | **p-value** |
| --- | --- | --- | --- |
| Number of admissions | 1446 | 591 |  |
| Age, years | 87 (85-88) | 87 (85-89) | 0.018 |
| Male gender | 646 (44.7) | 262 (44.3) | 0.887 |
| Able to live at home | 1225 (85.0)^a^ | 61 (84.7)^b^ | 0.947 |
| Able to move indoors | 1357 (93.9)^c^ | 40 (95.2))^d^ | 0.722 |
| Able to walk 400 meters | 971 (67.2)^e^ | 22 (71.0)^f^ | 0.662 |
| Able to climb stairs | 988 (68.3)^g^ | 13 (65.0)^h^ | 0.751 |
| Able to dress themselves | 1291 (89.4)^i^ | 29 (82.9)^j^ | 0.217 |
| Able to get out of bed | 1353 (94.0)^k^ | 33 (91.7)^l^ | 0.557 |
| Independent in ADL | 861 (59.5)^m^ | 319 (58.5)^n^ | 0.682 |
| Good PFS, n (%) | 705 (48.8) | missing |  |
| Admission type |  |  | <0.001 |
| Scheduled surgical | 298 (20.6) | 75 (12.7) |  |
| Emergency surgical | 380 (26.3) | 165 (27.9) |  |
| Medical admission | 768 (53.1) | 351 (59.4) |  |
| Diagnostic categories |  |  | <0.001 |
| Cardiac or vascular surgery | 323 (22.3) | 90 (15.2) |  |
| Gastrointestinal surgery | 236 (16.3) | 62 (10.5) |  |
| Neurological/neurosurgical diseases | 92 (6.4) | 67 (11.3) |  |
| Trauma | 113 (7.8) | 49 (8.3) |  |
| Other surgery | 40 (2.8) | 35 (5.9) |  |
| Cardiovascular diseases | 330 (22.8) | 160 (27.1) |  |
| Respiratory diseases | 132 (9.1) | 60 (10.2) |  |
| Metabolic disturbances | 154 (10.7) | 51 (8.6) |  |
| Intoxication | 7 (0.5) | 4 (0.7) |  |
| Miscellaneous | 19 (1.3) | 13 (2.2) |  |
| SAPS II | 39 (31-49) | 44 (34-57) | <0.001 |
| SAPS II without admission type | 33 (26-43) | 37 (29-51) | <0.001 |
| SOFA24 | 6 (4-8) | 7 (4-9) | 0.003 |
| TISS | 28.0 (22.8-33.8) | 27.7 (22.0-32.8) | 0.053 |
| LOS ICU | 1.54 (0.90-2.87) | 0.99 (0.58-2.07) | <0.001 |
| Treatment restrictions | 296 (20.5) | 182 (30.8) | <0.001 |
| ICU mortality | 93 (6.4) | 112 (19.0) | <0.001 |
| Hospital mortality | 249 (17.2) | 205 (34.7) | <0.001 |
| 1-year mortality | 555 (39.8) | 341 (59.7) | <0.001 |

Data for continuous variables are presented as median values (interquartile ranges), and data for categorical variables are presented as numbers of cases (%). SAPS II: Simplified Acute Physiology Score; SOFA: Sequential Organ Failure Assessment score, based on the first 24 hours; TISS: mean daily Therapeutic Intervention Scoring System 76 score; LOS ICU: length of stay (days) in intensive care unit; good PFS: good premorbid functional status (a person who can perform ADL and climb stairs without assistance).

Data available for, % a99.7, b12.2, c99.9, d7.1, e99.9, f5.2, g100, h3.4, i99.9, j5.9, k99.5, l6.1, m100, n92.2
