# Supplementary material for: Premorbid functional status as an outcome predictor in intensive care patients aged over 85 years
Source: BMC Geriatr. 2022 Jan 10;22:38. doi: 10.1186/s12877-021-02746-1 (PMC8751370; doi:10.1186/s12877-021-02746-1)
Supplement: Supplementary file 3 — Additional file 3. Factors predicting orders to restrict treatment activity [file 12877_2021_2746_MOESM3_ESM.docx]

**Supplementary table 3 Factors predicting orders to restrict treatment activity**

| **Factors** | **Adjusted OR** | **95% CI** | **p-value** |
| --- | --- | --- | --- |
| Type of admission |  |  | <0.001 |
| Scheduled surgical | reference |  |  |
| Emergency surgical | 7.01 | 3.96-12.42 |  |
| Medical | 11.53 | 6.66-19.95 |  |
| SAPS II without admission type^a^ | 1.04 | 1.04-1.05 | <0.001 |
| Age^b^ | 1.09 | 1.04-1.13 | <0.001 |
| Poor premorbid functional status | 3.09 | 2.33-4.08 | <0.001 |

^a^For each point, ^b^for each year of age.
