# Supplementary material for: Premorbid functional status as an outcome predictor in intensive care patients aged over 85 years
Source: BMC Geriatr. 2022 Jan 10;22:38. doi: 10.1186/s12877-021-02746-1 (PMC8751370; doi:10.1186/s12877-021-02746-1)
Supplement: Supplementary file 4 — Additional file 4. Treatment intensity, based on recorded Therapeutic intervention scoring system items [file 12877_2021_2746_MOESM4_ESM.docx]

**Supplementary table 4 Treatment intensity, based on recorded Therapeutic intervention scoring system items**

| **Intervention** | **85 years and older** |
| --- | --- |
| **Mechanical ventilation** | 1272 (62.4) |
| Controlled ventilation with or without positive end-expiratory pressure | 373 (18.3) |
| Controlled ventilation with intermittent or continuous muscle relaxants | 59 (2.9) |
| Intermittent mandatory ventilation or assisted ventilation | 839 (41.2) |
| Continuous positive airway pressure | 385 (18.9) |
| **Vasoactive treatment** ^a^ | 1442 (70.8) |
| Vasoactive drug infusion (1 drug) | 1207 59.3) |
| Vasoactive drug infusion (> 1 drug) | 516 (25.3) |
| Continuous antiarrhythmia infusions | 257 (12.6) |
| **Invasive monitoring** | 1875 (92.0) |
| Pulmonary artery catheter | 281 (13.8) |
| Intracranial pressure monitoring | 10 (0.5) |
| Measurement of cardiac output by any method | 341 (16.7) |
| Central venous pressure monitoring | 1044 (51.3) |
| Arterial line | 1860 (91.3) |
| **Haemodialysis** | 63 (3.1) |

Data is presented as numbers of cases (%). ^a^ Items were recorded for each ICU day, and therefore the sum of percentages may exceed 100%.
